# The Finance of a Year's Red Cross Work

**Published:** 1916-01-08

**Authors:** 


### THE FINANCE OF A YEAR'S RED CROSS WORK.

### The Administration of Two Millions of Money.

It was on October 20, 1914, that the British Red Cross Society and the Order of St. John of Jerusalem agreed to combine their forces and work hand in hand under a Joint War Committee of the two societies. When one realises the very complex character of the financial side of the work of such a gigantic organisation, congratulations will be readily offered to the Joint Finance Committee on the feat of producing the Report of the first working twelve months within sixty days of the close of the financial year.

It is true that in the volume of eighty-five pages, sixty-four of which are devoted to accounts and tables, no attempt has been made to refer to the individual worker; except so far as important figures require explanation, the task of publishing names of donors and subscribers has not at the present time yet been attempted. The Report states this aspect of the labours of the organisation in these words: "It may be thought that this Report is lacking in adequate expression of the obligation we are under to all who have enabled the work to be carried out, whether by their gifts or by their personal service, or by both. Of set purpose we have refrained from attempting to place our thanks on record. To do so would add pages to this Report, for the name of our helpers is legion. But there is an even stronger reason for remaining silent. We believe that no one desires to be thanked, and that all are proud and content to have the opportunity of doing what lies in their power to lessen the sufferings of the sick and wounded."

### RECEIPTS.

The total amount received and accounted for by the Joint Committee as shown in the Report is £1,864,036, and of this sum there remained on balance on October 20, 1915, £221,764. The war started on August 4, 1914, and from that time until the date of the amalgamation the two societies carried on their full work with separate organisation, the cost of the operations not being included in the foregoing figures. For some purposes, outside the scope of the work by arrangement jointly administered, the societies have continued their labours, and, of course, have preserved their identity. But together with the joint accounts is presented the individual statement of the British Red Cross Society, which shows the receipt of £299,574 and the expenditure of £176,046 in the first twelve months of the war. In addition, in reference to the joint accounts there are gifts and services of important accountancy value, such as the special exemption from taxation granted by the Government in respect of imported automobile parts; the practically free housing of the headquarters of the organisation through the kindness of the Royal Automobile Club, who granted free use of the offices to the end of 1914, and of Lord Michelham, who defrayed the balances of charges under this heading up to October 20, 1915; and free telegraph service to the East provided by the Eastern Telegraph Company. We select these items with accountancy value (which, perhaps, for the reason that they were not easily stated in cash, have not been included in the accounts) because others, in the shape of gifts rather than forms of service, find their correct places on the expenditure side of the financial statements. The latter come under the heading of "Stores," and as these gifts in kind amount to 43 per cent. of the total value handled (£463,455), not so to include them would have the effect of producing an erroneous picture of the financial position. The total value of these gifts in kind was £199,000, and, curiously enough, the value of the stores unissued on October 20 was £191,000. From the last fact one would judge that unpreparedness is not one of the vices of the Joint Finance Committee. To sum up the total receipts, and to justify our headline, it can safely be stated that a total sum of over two millions of money is represented by the administrative work described in the pages of the Report.

In approximate figures, too, it may be said that £2,000,000 has been expended on the current cost of Red Cross work. Assets there are, but these plainly are not of capital value, and there is little in the possession of the Joint Committee that a financier would consider of the nature of adequate security against debentures. This fact illustrates the difference between Red Cross and voluntary hospital work; the one has to provide for what everybody hopes is a comparatively brief period, the other for many years. Permanency in the matter of buildings, to some extent in respect of equipment, is not a feature of war work, although in some directions, such as hospital trains, ambulances, instruments for diagnostic and treatment purposes, the best that money can buy is rightly provided. This fact was brought home to us in a recent visit to that marvel of adaptation, King George Hospital, where we noticed with satisfaction the simple, yet, in the circumstances, adequate, character of the ward furniture and kitchen apparatus, as compared with the elaborate and valuable equipment of the *x*-ray department.

### EAR-MARKING CONTRIBUTIONS.

When people provide money to forward the activities of the Joint War Committee, they often ear-mark their contributions to provide that some particular branch of work shall be supported by their help. The feeling which actuates this allocation is understandable and praiseworthy. It has, however, as the Report rather plaintively points out, a certain disadvantage; work cannot be delayed, and lives possibly endangered, because one fund is richly supplied while another has been temporarily neglected. The only way to meet the difficulty is for one fund or one branch to support another, and this practically amounts to the same thing as if all the moneys had been paid into a general account, and all the expenditure met from the gross assets. The difficulty, which, after all, is one of the perplexities of accountancy, is instanced by overdrawn balances of the Transport of Wounded Fund and the Netley Maintenance Account.

But, on the other hand, some prefer to give money in this way, and an efficacious encouragement of almsgiving cannot be rejected. For instance, the British Farmers' Red Cross Fund, raised through a large number of individual appeals to the agricultural interests throughout the kingdom, was given to finance the Enteric Hospital at Calais. This leads us to another point, to a feature of the administration which must please the purist in charity finance: we allude to the clear statement that the cost of raising money through subsidiary appeals, naturally often at a higher rate than the cost of the same work as applied to the main fund, is included on the expenditure side of the account. Another businesslike feature is that all known liabilities have been introduced into the income and expenditure accounts, although their discharge was subsequent to the issue of the statement.

### Management Expenses.

The Report states that "the management expenses (including the cost of receiving, handling, distributing, and recording at head office, the whole of the funds received by the Joint Committee) would amount to £21,221 6s. 3d., representing 1¾ per cent., or 2¾d. in the pound of the whole of the income, or if we add the cost of appeals (£6,137 0s. 11d.), 1.46 per cent., or 3½d. in the pound. These figures we regard as eminently satisfactory. That they should be so low is due largely to the fact that so many of our workers are unpaid. We have no head of a department in receipt of a salary." But are they low? It is difficult to say if they are low or high because there is hardly anything of a similar nature to compare them with. Certainly if one examines detail no form of extravagance is apparent, although ninety-four paid and thirty-one voluntary workers for a department dealing with finance alone appears to be an ample staff. The salaries paid, bearing in mind the fact that the posts are temporary, are not excessive, only fourteen persons receive over £150 a year, none over £500.

In the stores department there are thirty-four voluntary and ninety-eight paid workers, and they have handled goods to the value of £463,455, at a cost of £13,712, or 2.96 per cent. of the gross.

### A Magnificent Record.

But these are details appealing to the business mind. For those who wish to form an impression of the general picture, in its full magnificence, the best way perhaps after all to regard work of essentially national character, "where the whole machinery had to be improvised at the outbreak of the war, and where developments of the work have been so many and so unexpected," is not to linger too long on such points but look more fixedly at the main operative features of an undertaking which has grown to such vast dimensions.

The meaning of the "far-flung battle-line" is brought home to a Briton to-day as it has never been before. The field in which our Red Cross is engaged, as revealed by this Report and chiefly illustrated by statistics and accounts (showing not only to the statistician how expressive figures can be), is wide indeed. France and Belgium, the Near East (including Egypt), Serbia, Montenegro, Italy, the Persian Gulf, each claims a large share of money and endeavour, and the effects of the great merciful enterprise are felt as far as Nairobi, the Cameroons, and Rhodesia. Wide though this field is, it may yet be more extended, for no man knows how far the flame of battle, burning so fiercely, may still reach. Generous as the response to the irresistible appeal has been, further large drafts on the generosity of the public must be made, and will, we are confident, be met with cheerfulness.

Necessarily, in the volume before us, ample though it is for the purpose it serves, the reader fails to find any attempt at detailed description of the manifold work of the Joint War Committee. We learn under main headings of the activities of the workers in Red Cross hospitals at home and abroad, of the transport of wounded, of the provision and equipment of hospital ships, of help to kindred institutions, of the endeavours to trace missing soldiers, of registration of the graves where our dead lie buried, of supplies sent to our men who are prisoners in the hands of the enemy, and of the many other phases of the work. The supreme interest attached to the main facts of all these activities will no doubt be satisfied in good time by an extended report of the operations of the great organisation. But we know that the work has all the characteristics of our nation: deliberate in its movement and, if slow, yet always attaining a momentum, secure and increasing in degree, which is in itself a presage of victory.

